# Diseases of the Upper Air Passages

**Published:** 1896-01-11

**Authors:** 


### DISEASES OF THE UPPER AIR PASSAGES.

**Tuberculosis.**—The diagnosis of tuberculosis of the nose, pharynx, and larynx is discussed by Clarence Rice,[1] who calls attention to many points worth bearing in mind. As regards the relative frequency with which the different portions of the region are affected, Willigk in 476 autopsies of tuberculosis found only one in which the nose was involved, and in 1,317 autopsies of similar cases found the larynx involved 237 times and the pharynx once, while Louis found four cases of pharyngeal disease in 120 patients. As regards the nasal affection then, it is obviously very rare, and is practically always secondary to pulmonary tuberculosis. It occurs in two forms, (*a*) ulceration, and (*b*) small nodular growths attached to the turbinated tissues. As to the diagnosis we should be suspicious of any chronic ulceration of the nose which resists the effects of iodide of potassium, and which is associated with tuberculous pulmonary disease. The secretions contain the bacilli. Rice commits himself to the usual contradiction in terms when he says, "Perhaps I ought to mention the possibility of the presence of lupus in the nose, which also is probably a tuberculous process, since the tubercle bacilli have often been found in the ulcerations of lupus. It is possible that some of the ulcerative cases which have been called tuberculosis of the nose have been lupus."

Tuberculosis of the pharynx is usually associated with acute miliary tuberculosis. Here tuberculous infiltration is probably always secondary to deposits elsewhere. The only form of ulceration of the pharynx at all likely to give rise to difficulty in differentiation is syphilis; but the tuberculous deposit and ulceration here, as in the larynx, presents the characteristic greyish-white surface, spreading superficially by the union of minute erosions, and the process is usually highly painful.

First, as regards the diagnosis of typical cases of laryngeal tuberculosis. Can any disease present appearances more easily recognised than that tumefaction of the larynx due to tuberculous infiltration, that pale, boggy, semi-opaque, partly œdematous, partly inflammatory swelling of the arytenoid cartilages which has given to them the name of "club-shaped," and to the epiglottis that of "turban-shaped"? We can think of nothing that such enlargements, if they are bilateral and involve both the epiglottis and the arytenoids, resemble, unless it be an œdema of the larynx; but a diagnosis between these need hardly be considered, because the cases are so widely different in their entire history, the one being an acute, and the other a chronic, process. When the tuberculous infiltration attacks only a single arytenoid, then the question will arise as to whether the process may not be rather a syphilitic one, or an idiopathic perichondritis, or possibly some variety of neo-plasm. Unilateral enlargement of the crico-arytenoid articulation is a very rare manifestation of tuberculosis.

Rice cites Solly,[3] of Colorado, who has found that in 250 cases of pulmonary tuberculosis there were 45 in which the larynx were involved; 25 of these had not broken down into ulceration at the time of the first examination. In his case the ulcerations were situated upon the vocal cords in 50 per cent., upon the arytenoids in 45 per cent., in the inter-arytenoid commissure and in the epiglottis in 30 per cent, and upon the false cords in 20 per cent.

As regards danger signals or earliest manifestations he refers to localised anæmias of the hard and soft palate in a pharynx which is otherwise healthy in aspect. With this pallor he has noticed enlarged capillaries merging from the different portions of the palate towards the uvula. The enlargement of the papillary layer in the inter-arytenoid commissure, however slight it may be, is also a suspicious sign. Feebleness of action of the transverse or arytenoid muscle or of the thyreo-arytenoid muscle are points to be considered. Rice recalls two cases in which simple warty papillomata existed in the larynx two and four years before tuberculous laryngitis appeared, and thinks it possible they may sometimes be of tuberculous origin. An unusual manifestation of tuberculous ulceration of the larynx is adhesion between approximating ulcerative surfaces.

Tuberculous neoplasms may be divided into three groups following J. Mackenzie—(*a*) Granular hyperplasias, (*b*) papillary excrescences, (*c*) smooth neoplasms. The granular hyperplasias, the most common, may sometimes be seen in quite a prolific state springing from the surface of ulcerations, and occluding the larynx to a marked degree; but it usually degenerates before any necessity to open the trachea occurs. These may occasionally resemble malignant growths, but there is a much stronger resemblance between malignant growths and syphilitic hyperplasias. As regards the wart-like excrescences, Störk considers them very characteristic of incipient tuberculosis, and says they are connective tissue hypertrophy in the neighbourhood of the congested arytenoids. The third form of growth is that described by J. Mackenzie in 1882, and of which Payson Clark has collected 42 cases, viz., tuberculous neoplasms.

Clark states that tuberculous disease of the larynx appears very rarely to manifest itself in the form of smooth, rounded tumours, single or multiple, which are found, on microscopic examination, to consist of tuberculous tissue. These growths appear beneath an unbroken surface, and rarely break down into an ulcerative condition, are usually sessile, and the colour of the overlying mucous membrane is not much changed. They resemble fibromata and sometimes papillomata, and may be strongly indicative of malignant disease. Rice says that it is interesting to note that in Dr. Mackenzie's case of tuberculous tumour of the trachea the patient died of carcinoma of the stomach, and that secondary cancerous deposits were found in many organs of the body, but that the lungs were tuberculous. The diagnosis of this case was made with the microscope, and the growth contained an aggregation of distinct tuberculous nodules. The mucous membrane covering the growth was unbroken; the case was supposed to be malignant until the microscope showed its true character.

**The Surgical Treatment of Laryngeal Tuberculosis** is a subject that has been well to the fore recently. Gleitsmann[4] summarises the present position in an admirable article. Surgical treatment, broadly speaking, is either endo-laryngeal or extra-laryngeal. Endo-laryngeal treatment comprises: (1) Incision with knives or properly curved scissors; (2) curettement;

(3) submucous injections; (4) electrolysis; (5) galvanocautery. Extra-laryngeal measures are: (1) Laryngotomy with excision of diseased part; (2) extirpation of the larynx; and (3) tracheotomy. Intubation has also been performed for laryngeal tuberculosis. Gleitsmann urges in favour of curettement, that though it cannot be expected that it will directly exert a favourable influence on the almost always present pulmonary complications, yet we are justified in speaking of a cure of the larynx when, in spite of the continuance of pulmonary disease, the largyngeal symptoms have subsided, when the larynx bears a normal aspect, and furthermore, when no trace of the disease is found at a post-mortem—conditions which are established beyond doubt and are enumerated in the literature. An argument against curettement is that relapses cannot be prevented, an assertion which cannot be contested. But with increased experience, and the endeavour to remove all diseased parts till healthy tissue is reached, the relapses with curettement will become less frequent, and the results will certainly compare favourably with other methods of treatment. One of the most distressing symptoms of laryngeal tuberculosis is dysphagia, caused by infiltration of the arytenoid region, and it is this particular feature of the disease which is best adapted for curettement. Being more frequently bilateral than unilateral, the removal of the hard, dense swelling by the Krause-Heryng double curettes can often be accomplished in one sitting. It is astonishing how quickly the wound heals. The suffering of such patients, due to more abundant nerve proliferation in tubercular disease, as demonstrated by Gougenheim and Balzer, is so intense, and the relief granted as a rule so great, that arytenoidectomy is in favour with most operators in cases in which otherwise curettement is contra-indicated, viz., in active pulmonary disease with hectic. Moreover, the patient, as the result of curettement, is able to take food freely, and surgical treatment is also followed by improvement of the voice, cough, and respiration, results which are the natural consequence of cicatrization, &c.

Summarising the indications for curettement, he gives the following conditions: (1) Cases of primary tuberculous affections without pulmonary complication. (2) In cases of concomitant lung disease, which is either in the incipient stage or has at least not progressed to softening, and hectic conditions. (3) It is best adapted for circumscribed ulcerations and infiltrations of the larynx especially. (4) For the dense, hard swelling of the arytenoid region, the ventricular band, the posterior wall, for tuberculous tumours, and for affections of the epiglottis. (5) In advanced lung disease, with distressing dysphagia resulting from direct infiltration of the epiglottis, curettement is justifiable as the quickest method of affording relief.

Contra-indications are: (1) Advanced pulmonary disease and hectic. (2) Disseminated tuberculous disease of the larynx, leaving little or no area of healthy tissue. (3) Extensive infiltrations, producing severe stenosis when tracheotomy is indicated. He will also not recommend surgical treatment to nervous, distrustful patients, who lack the necessary perseverance or confidence in their physician.

Gleitsmann gives his own experience in twelve patients, of whom all had pulmonary complication. There were two operations for infiltrations of the posterior laryngeal wall alone, one for such with affection of the ventricular band combined, four arytenoidectomies, three arytenoidectomies and excision of the ventricular band, and two of the latter alone. Arytenoidectomy had to be performed a second time on two patients on account of recurring infiltration. One patient died from heart failure, another from his advanced pulmonary disease. Four patients are without recurrence of laryngeal disease from six to ten months afterwards, one of whom had affection of the posterior laryngeal wall, another of the ventricular band, and two of the arytenoid region.

[1] N. Y. Med. Journ., Oct. 19, 1895. [2] Section sergebnisse an der Prager path-anat., Anstalt, Feb. 1, 1850, to Feb., 1852. [3] Ther. Gaz., Nov. 15, 1893. [4] N. Y. Med. Journ. Oct. 19, 1895.

